# Patterns of Circle of Willis Variants among Patients Undergoing 3 Tesla Magnetic Resonance Angiography at a Tertiary Care Hospital: An Observational Study

**DOI:** 10.31729/jnma.9057

**Published:** 2025-06-30

**Authors:** Subash Thapa, Rabindra Desar, Jorina Basnet, Sushmita Thapa

**Affiliations:** 1 Department of Radiodiagnosis, Nepal Police Hospital, Mahrajgunj, Kathmandu, Nepal; 2 Department of Radiodiagnosis, Civil Service Hospital, Minbhawan, Kathmandu, Nepal; 3 Department of Internal Medicine, KIST Medical College & Teaching Hospital, Imadol, Lalitpur, Nepal; 4 Department of Accident and Emergency, Birendra Hospital, Chhauni, Kathmandu, Nepal

**Keywords:** *circle of willis*, *magnetic resonance angiograpgy*, *variants*

## Abstract

**Introduction:**

The Circle of Willis (CoW) is a crucial cerebral arterial network, and its anatomical variations may influence cerebrovascular health. This study aimed to determine the prevalence and patterns of CoW variants using 3 Tesla magnetic resonance angiography.

**Methods:**

This retrospective cross-sectional study was conducted at the Department of Radiology, of a tertiary care center, Nepal, from October 2022 to September 2024 following ethical approval (IRC- 16/2024). Inclusion required adequate 3T MRI imaging using a TOF MRA protocol. (TR 20 ms, TE 3.5 ms, flip angle 15°, slice thickness 0.5 mm). Patients with cerebrovascular disease, prior interventions, or contraindications were excluded. Circle of Willis morphology was analyzed for prevalence, variants, and age/gender correlations using SPSS and Excel.

**Results:**

The study included 384 participants of which 206 (53.65%) were male. The mean age was 59.20 ± 16.30 years. A complete Circle of Willis (COW) was observed in 139 (36.19%), while 220 (57.39%) were incomplete. Anterior anomalies 126 (32.81%) primarily consisted of hypoplasia, 95 (75.40%) right A1 dominance and absence 24 (19.05%). Posterior variants 345 (89.84%) involved hypoplasia 155 (44.93%) or absence 189 (54.78%) of the posterior communicating artery, with fetal PCA origin in 121 (31.51%).

**Conclusions:**

This study found common Circle of Willis variations, especially in older adults. Hypoplasia of the posterior communicating artery was the most frequent variation, with differences observed by gender.

## INTRODUCTION

The Circle of Willis (CoW) is an arterial network at the brain's base that ensures collateral circulation and protects against ischemia.^1^ Comprising the anterior and posterior cerebral arteries, anterior communicating artery, and posterior communicating arteries, it plays a vital role in maintaining cerebral blood flow.^1-3^ Anatomical variations are common and effectively demonstrated by imaging modalities like magnetic resonance angiography (MRA), with clinical relevance in cerebrovascular events.^2,5^

Although CoW morphology has been studied in various populations,^6-8^ few have focused on the Nepali population.^9,10^ Genetic, environmental, and imaging differences contribute to inconsistent prevalence rates^6,11,12^ Understanding regional variations can aid clinical decision-making and cerebrovascular care.

This study utilizes 3 Tesla MRA for high-resolution, non- invasive evaluation of CoW morphology, offering precise detection of arterial variants.^10,13,14^ Data from a tertiary center in Kathmandu aim to enhance regional and global neurovascular understanding.^15,16^ Establishing a reference dataset will improve insights into vascular anatomy and guide stroke prevention strategies.^11,12,15,17^

## METHODS

This study was a retrospective, descriptive, cross-sectional analysis conducted at the Department of Radiology, Civil Service Hospital, Nepal. The study evaluated MRA images of patients referred for brain imaging over a two-year period, from October 2022 to September 2024. Ethical approval was obtained from the Institutional Review Committee of Civil Service Hospital (IRC Protocol No. 16/2024).

The study included patients aged 18-80 years who were referred for MRA of the brain during the study period. Patients with adequate imaging quality obtained using a 3 Tesla MRI scanner were included.

Exclusion criteria consisted of patients with significant cerebrovascular diseases, such as major ischemic infarcts or acute intracranial hemorrhages, a history of neurosurgical or head and neck interventions, or incidental brain pathologies, including aneurysms, arteriovenous malformations, or brain tumors. Patients undergoing repeat imaging or those with implanted devices contraindicated for MRI, such as pacemakers or ferromagnetic aneurysm clips, were also excluded.

The required sample size was calculated using the formula for cross-sectional studies:

Sample size (n) =Z^2^ * p(1-p)/E^2^

where,

Z = 1.96 for 95% confidence level,

p = Expected prevalence (50% assumed for maximum variability),

E = Margin of error (5% or 0.05).

Based on this calculation, the sample size was determined to be 384 participants.

Imaging was performed using a 3 Tesla MR scanner (Siemens Magnetom Vida, Germany) equipped with a standard head coil. The time-of-flight (TOF) MRA protocol was used with the following parameters: repetition time 20 ms, echo time 3.5 ms, flip angle 15 degrees, slice thickness 0.5 mm, and a matrix size of 256. Flow was oriented from feet to head, with a saturation band applied at the head end to minimize artifacts. Imaging quality and resolution were optimized to visualize the intricate arterial structures of the Circle of Willis.

Data were collected retrospectively from the hospital's radiology records. Consecutive patients meeting the inclusion criteria were enrolled, and their imaging data were anonymized for analysis. MRA images were acquired using the Time of flight (TOF) technique, which provides non-contrast visualization of cerebral arteries. All imaging parameters were standardized to ensure consistency and reliability. Demographic variables, including age and gender, were recorded. The dependent variables included the presence of anatomical variations in the Circle of Willis, categorized as complete or incomplete configurations and further subclassified into anterior and posterior circulation variants.

The anterior circulation includes the intradural internal carotid artery (ICA) and its branches, along with the anterior cerebral artery (ACA), middle cerebral artery (MCA), anterior communicating arteries (ACoAs), and posterior communicating arteries (PCoAs). The posterior circulation consists of the vertebrobasilar trunk, its branches, and the terminal bifurcation into the two posterior cerebral arteries (PCAs).^18^

Anterior circulation variants include absent, hypoplastic (<0.8 mm), fenestrated (Separate origins with no downstream convergence), duplicated arteries, azygos ACA (single A2 trunk), and bihemispheric ACA (hypoplastic A2 with contralateral A2 dominance). Posterior circulation variants include absent, hypoplastic, fenestrated, duplicated arteries, and fetal origin of PCA (hypoplastic P1 with prominent ipsilateral PCom supplying P2).^18^

All MRA images were analyzed using Siemens Health Care Syngo.via software on EIZO high-resolution color monitors. Two experienced radiologists, each with more than seven and ten years of expertise in neuroimaging, independently reviewed the scans. Arteries with a diameter of >0.8 mm were classified as normal, while those <0.8 mm were categorized as hypoplastic. Non-visualized or discontinuous vessels were classified as absent. Discrepancies in interpretations were resolved through consensus.

Data were analyzed using Microsoft Excel (Microsoft Office 2019) and SPSS version 20.0. Descriptive statistics, including frequencies and percentages, were used to report the prevalence and patterns of Circle of Willis variants.

## RESULTS

**Figure 1:**
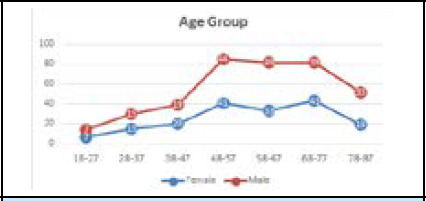
Case distribution by age group and gender (n=384).

Out of 384 participants 178 (46.35%) were females, and 206 (53.65%) were males with a mean age of 59.20 ±16.30 years (range: 18-87 years). Age distribution revealed the highest representation in the (48-57) age group 85 (22.14%). (Table 1 and Figure 1).

**Table 1:** Age and Gender Distribution (n = 384).

Age Group	Female	Male	Total (%)
18-27	7	7	14(3.64%)
28-37	15	15	30(7.81%)
38-47	20	19	39(10.15%)
48-57	41	44	85(22.14%)
58-67	33	49	82(21.35%)
68-77	43	39	82(21.35%)
78-87	19	33	52(13.54%)
Total	178	206	384

A complete Circle of Willis (COW) was observed in 139 (36.19%) of cases, while 220 (57.29%) had incomplete configurations. A structurally normal COW, defined as anatomical completeness without hypoplasia, absence, or duplication, was rare 25 (6.51%). Posterior circulation involvement dominated incomplete COW cases, followed by combined anterior- posterior variants 120 (54.55%) (Table 2).

**Table 2:** Variants of Circle of Willis (CoW) (n = 384).

Variations	n(%)
Complete Circle of Willis	139(36.2%)
Incomplete Circle of Willis	220(57.29%)
Structurally normal CoW	25(6.51%)
Anterior Circulation Variants	126(32.81%)
Bilateral A1 Hypoplastic	4(1.04%)
Right A1 Hypoplastic	59(15.36%)
Left A1 hypoplastic	32(8.33%)
Right A1 Absent	18(4.69%)
Left A1 Absent	6(1.56%)
Right A1 Fenestration	6(1.56%)
Left A1 Duplication	1(0.26%)
Azygos ACA	7(1.82%)
Bihemispheric ACA	1(0.26%)
Trifurcation ACA	1(0.26%)
Posterior Circulation Variants	229(59.64%)
Hypoplastic Pcom or PCA	95(24.74%)
Bilateral Pcom Hypoplastic	48(12.5%)
Right Pcom Hypoplastic	18(4.69%)
Left Pcom Hypoplastic	29(7.55%)
Right P1 Hypoplastic	26(6.77%)
Left P1 Hypoplastic	8(2.08%)
Bilateral P1 Hypoplastic	26(6.77%)
Bilateral Pcom Absent	97(25.26%)
Right Pcom Absent	35(9.11%)
Left Pcom Absent	48(12.5%)
Bilateral P1 Absent	3(0.78%)
Right P1 Absent	3(0.78%)
Left P1 Absent	3(0.78%)
PCA duplication	1(0.26%)
Fetal origin Right PCA	62(16.15%)
Fetal Origin Left PCA	26(6.77%)
Fetal origin PCA Bilateral	33(8.59%)

Anterior circulation variants were identified in 126 (32.81%) of cases. Hypoplasia was the most common anomaly 95 (75.40%) , predominantly affecting the right A1 segment 59 (62.11%). Absence of the A1 segment occurred in 24 (19.05%), with right-sided absence 18(75%) exceeding left-sided 6 (25%). Rare findings included fenestration 6 (4.76%), duplication 1 (0.79%), and accessory variants: azygos ACA 7 (5.56%) and bihemispheric ACA 1(0.79%).

Posterior circulation variants were more prevalent 345 (89.84%) than anterior anomalies. Hypoplasia 155 (44.93%) and absence 189 (54.78%) were predominant. The posterior communicating artery (Pcom) was most frequently involved: bilateral Pcom hypoplasia 48 (33.10%) and absence 97 (66.90%). Fetal origin of the PCA was observed in 121 (31.51%) of cases, with right fetal (RTF: 62 (51.24%) exceeding bilateral BLF: 33 ( 27.27%) and left fetal LTF: 26 (21.49%) origins.

## DISCUSSION

The Circle of Willis (CoW), formed by internal carotid and vertebral arteries, maintains collateral circulation. Its anatomical variations hold clinical relevance in stroke, aneurysms, and cerebrovascular diseases.^4,5,17^

In our study of 384 participants (178 females and 206 males), the demographic distribution revealed that the majority of individuals with abnormal Circle of Willis (CoW) configurations were within the 58-67 and 68-77 age groups. This finding is consistent with the study by De Silva KrD et al.,^6^ which also reported a higher prevalence of CoW variations with increasing age.

In the current study, 36.19% of participants exhibited a complete Circle of Willis (CoW), whereas 220 (57.29%) had incomplete configurations.

In this study, 36.19% of participants in the current study showed a complete Circle of Willis (CoW), while 220 (57.29%) had incomplete configurations. These findings are comparable to those of Kumari et al.,^17^ who reported 44% complete and 56% incomplete CoWs. Additionally, studies utilizing cadaveric dissections and imaging modalities have documented varying rates of completeness, ranging from 14.20% to 44%,^6,9,13,17^ highlighting notable variability across different populations and methodologies. This discrepancy may be attributed to differences in sample populations, as our hospital-based cohort likely included a higher proportion of individuals with cerebrovascular risk factors. Notably, the proportion of structurally normal CoWs in our study (6.51%) closely aligns with previous reports, which observed truly normal configurations in only 7-11% of cases,^9,13,19^ reinforcing the rarity of a perfectly formed CoW.

The most common variant observed in CoW incompleteness was in the posterior circulation (70%), consistent with several reports indicating that the posterior segment is more prone to anomalies than the anterior region.^2,6,7,11,17^ Posterior communicating artery (PCoA) hypoplasia was the most frequently observed anomaly.^4^-^6^-^8^ with bilateral aplasia observed in 18.40% of cases, a finding that aligns with the results reported by Hashem et al.^12^

The prevalence of fetal origin of the posterior cerebral artery (PCA) in the present study was 121 (31.51%) with the right fetal origin (RTF) being the most common (51.24%), followed by bilateral fetal origin (BLF: 27.27%) and left fetal origin (LTF: 21.49%). This finding is consistent with Karatas et al.,^20^ who reported a fetal PCA prevalence of 30.20%, with a similar predominance of right-sided fetal origin. Notably, the higher prevalence of RTF in our study aligns with the findings of Sharma et al.,^10^ which documented 53.50% right fetal origin, reinforcing the notion that right-sided dominance is a consistent anatomical variant across populations. The presence of fetal PCA is clinically significant due to its association with altered hemodynamics and potential implications for ischemic stroke.^9^

The anterior circulation of the Circle of Willis (CoW) demonstrated significant anatomical variability in this study, with variants observed in 126 (32.81%) of cases. Hypoplasia was the most frequent anomaly, accounting for 95 (75.40%) cases, predominantly affecting the right A1 segment (62.1%). Additionally, the absence of the A1 segment was found in 24 cases (19.05%), with right-sided absence (75%) being more prevalent than left-sided absence (25%). Less common anomalies included fenestration (4.76%), duplication (0.79%), and accessory variants such as azygos anterior cerebral artery (ACA) (5.56%) and bihemispheric ACA (0.79%).

These findings are consistent with several previous studies.^1,17^ One study by De Silva KrD et al.,^6^ reported A1 hypoplasia in 4.10% of cases, which is significantly lower than the prevalence observed in our study. In contrast, a study by Kumari et al.,^17^ identified anterior circulation variations in 48% of cases, which is higher than our findings, suggesting potential demographic or methodological influences. The relatively rare variants of anterior circulation, including azygos ACA (5.56%) and fenestration, were identified in our study, consistent with other studies that consider these anomalies rare across different populations.^16^

This study has several limitations. As a retrospective analysis, it relied on existing records, limiting control over data completeness and potentially missing clinical details. The sample was drawn from a single tertiary care center (Civil Services Hospital), which may introduce selection bias and limit the generalizability of findings to the broader central Nepali population. Additionally, the focus on a specific regional group may not reflect variations present in other ethnicities or populations with differing genetic and morphological characteristics. Future research should explore the implications of these variations on stroke risk and cerebrovascular outcomes in diverse populations.

## CONCLUSIONS

This study provides valuable insights into the prevalence and patterns of Circle of Willis (CoW) variations in the central Nepali population. CoW anomalies were most prevalent in older age groups (58-77 years). Structurally normal CoW, was less prevalent aligning with previous studies that highlight its rarity. The posterior circulation was the most affected region with hypoplasia being the most frequent anomaly, predominantly in the right A1 segment.
